# Performance Analysis of Troposphere Scattering Communication Channel with Chirp-BOK Modulation

**DOI:** 10.3390/e26121052

**Published:** 2024-12-04

**Authors:** Junhu Shao, Zaiping Liu, Yishuo Liu, Tianjiao Xie

**Affiliations:** 1 Xi’an Key Laboratory of Wireless Optical Communication and Network Research, School of Automation and Information Engineering, Xi’an University of Technology, Xi’an 710048, China; jhshao@xaut.edu.cn (J.S.); 2220321179@stu.xaut.edu.cn (Z.L.); 2220320071@stu.xaut.edu.cn (Y.L.); 2 China Academy of Space Technology (Xi’an), Xi’an 710100, China

**Keywords:** troposcatter, chirp signal, multipath channel, bit error rate

## Abstract

By utilizing chirp-BOK (binary orthogonal keying) modulation into a troposphere scattering communication system, a lower demodulation threshold can be achieved with excellent linear frequency modulation properties in a strong noise and weak signal environment. Firstly, the bit error rate (BER) formula of chirp-BOK modulation over a Rayleigh fading channel was derived theoretically. Then, the BER performance with different chirp-BOK parameters were numerically calculated. In order to investigate the performance of chirp-BOK over deeping fading troposphere scattering link, a seven-path equal-delay Rayleigh fading model was employed. Finally, the system BER performance was simulated under different tap delay and time–bandwidth product parameters. The results demonstrate that when BER reaches $10^{-4}$, the optimal configuration of the system achieves a gains approximately from 1.7 dB to 10 dB compared to non-optimized configuration under different Path-Gain-Vector with varying tap delays.

## 1. Introduction

Troposphere scattering communication is a form of wireless link that utilizes atmospheric inhomogeneity to scatter or reflect ultra-short and short waves, which makes over-the-horizon (OTH) communication possible [1]. This method has become an essential component of the military communication technique due to its characters, such as long transmission range, low susceptibility to channel medium damage and good anti-jamming capabilities. However, a major drawback is the large signal attenuation and deep fading during the troposcatter channel, resulting in adverse multipath interference on the system performance.

Chirp spread spectrum (CSS) technology is an optional scheme in the IEEE802.15.4a physical layer standard [2]. It is a non-single-frequency wave modulation technique that uses chirp signals with different frequencies to carry information. These signals have good cross-correlation characteristics and are less sensitive to environmental noise. Additionally, chirp signals can have an effect of energy compression after matched filtering, which leads to a good performance against multipath fading and Doppler shift even at very low transmit power levels.

Winklery firstly applied CSS technology to the field of wireless communications by using chirp up-sweep and down-sweep signals to transmit binary data [3], i.e., binary orthogonal keying (BOK) modulation. Lee et al. applied the chirp-BOK scheme to transmit information in the ultrasonic-frequency band and achieved reliable long-distance transmission in severely frequency-selective fading channels [4]. Currently, CSS communication has been widely studied within two categories: modulating baseband information into the parameters of the chirp signal itself [3,5,6,7], or directly utilizing the chirp signal as a carrier for digital modulation [8,9]. Refs. [10,11] introduced the modulation and demodulation process of chirp signal, but they lacked the description of rigorous mathematical signal processing. Ref. [7] presented the mathematical formulas for modulation and demodulation process of frequency shift chirp modulation (FSCM), providing a theoretical derivation of optimal receiver with low complexity based on FFT. Ref. [12] analyzed the technical characteristics and performance of FSCM demodulation under the residual frequency offset. Ref. [13] proposed a fractional chirp rate grounded on CSS (FCR-CSS) as a physical layer-inspired approach to enhance the capacity of CSS modulation based on IoT networks. Ref. [14] developed a novel OFDM-chirp modulation scheme with a low peak-to-average power ratio (PAPR). An analysis method was introduced to evaluate the corresponding symbol error rate (SER) under carrier frequency offset (CFO) in a Rayleigh fading channel. Ref. [15] designed a non-coherent distributed transmission (NCDT) CSS system for low-power communication. Ref. [16] proposed a two-dimensional modulation method chirp-MFCSK for the LoRa system. Ref. [17] raised phase shift keying based on a CSS (PSK-CSS) multiple-access system with a single chirp rate. On the ground of the analysis results under multiple access interference, the PSK-CSS system can support synchronous and asynchronous transmissions simultaneously by multiplying different random phases to the data symbols of different users.

Owing to the special demands and characteristics of the tropospheric scattering communication system, the channel modeling and the adaptive reliable transmission technique have always been a research hotshot. Ref. [18] provide a reference for the theoretical characteristics of communication channels. Ref. [19] presented the troposcatter propagation principles along with several mathematical models, demonstrating integrated theoretical analysis with experimental measurements. Ref. [20] tested the fading characteristics of scattered signals and established a signal model with radar parameters, finally proposing a suitable beamforming and detection algorithm. In ref. [21], equalization techniques were incorporated into a scattering communication system to mitigate inter code interference and achieved high-speed transmission. Ref. [22] investigated the impact of atmospheric conditions on signal transmission and provided insights into uncertainty estimation, serving as a reference for an alternative statistical channel model for bit error rate (BER) analysis. Ref. [23] proposed an innovative anti-fading method for tropospheric scattering communication based on frequency hopping, which also explored synchronization technologies to improve the system performance.

In this paper, by utilizing a chirp modulation scheme into a troposphere scattering communication system, the mathematic formulation of a chirp-BOK signal was introduced to describe the characteristics of the signal passing through troposcatter channels. Then, the averaged BER formula was theoretically deduced over a Rayleigh flat fading channel and frequency-selective fading channel, respectively. In order to investigate the suitable chirp-BOK parameters to deal with different fading parameters, a seven-path fading channel model was established with tap delays and multipath gains. Finally, the BER performance of the chirp-BOK system was simulated and analyzed, with the results showing that an adaptive optimal chirp configuration could offer some significant performance gains.

The main contributions of this work are twofold. Firstly, the theoretical averaged BER formula of chirp-BOK modulation over a troposcatter fading channel was deduced and shown, and it was consistent with the corresponding simulation results over the established seven-path fading model. Secondly, for a troposcatter communication environment with a Doppler shift, multipath gain vectors and delays, optimal parameter configurations for chirp-BOK modulation were achieved to obtain some significant performance gains.

## 2. System Model with Chirp-BOK Modulation

**Figure 1:**
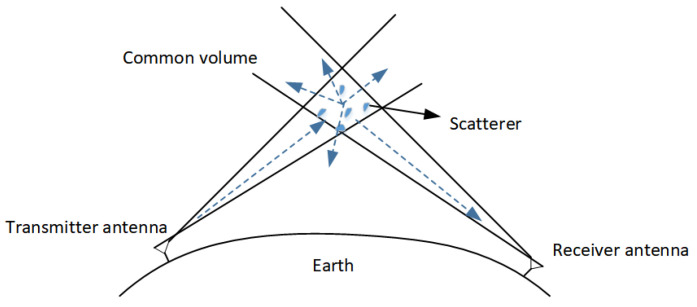
Troposcatter propagation path.

The schematic diagram of tropospheric scatter propagation is shown in Figure 1, with the transmitter and receiver far apart. In Figure 1, the “scatterers” in the troposphere can form a scattering effect on electromagnetic waves in the microwave band, thereby realizing OTH communication. The transmitter sends the signal toward the troposphere without being blocked by terrestrial obstacles. Although most of the transmitted signal passes out of the troposphere, the remainder scatters at the troposphere, which we call “common volume”. In order to set up a tropospheric scatter communication link, an antenna facing a horizontal line should be placed with an elevation angle as low as possible to minimize the path length. As shown in Figure 1, a common volume is formed at the intersection of the antenna beam widths, which can scatter the radio waves to the receiver.

### 2.1. Chirp Signal Model

Within a symbolic period, a chirp signal can be expressed as

(1) $$s\left(t\right)=a\left(t\right)e^{j\left(2\pi f_{0}t+\pi \mu t^{2}\right)},0\leq t\leq T_{b},$$

where a(t) represents the chirp signal envelope, which is usually a rectangular pulse; $f_{0}$ is the initial frequency; μ is the chirp rate, which denotes the instantaneous frequency change rate of the chirp signal; $T_{b}$ denotes the duration of the chirp signal; and $B=\left|\mu \right|T_{b}$ is used to represent the chirp signal bandwidth. When μ>0 or μ<0, the frequency of the chirp signal increases or decreases linearly during the sweep time, which is called the up-chirp signal or down-chirp signal.

A matched filter is a linear filter in which the ratio of signal power to noise power at the output is maximized, and receivers use matched filters to detect received signals. The optimal matched filter for an up-chirp signal is a down-chirp filter, and vice versa. Taking the up-chirp signal as an example, the output waveform of the signal after the matched filter is

(2) $$y_{1}\left(t\right)=\sqrt{BT_{b}}\cdot \frac{\sin\left[\pi BT_{b}\left(1-\frac{\left|t\right|}{T_{b}}\right)\right]}{\pi BT_{b}}\cdot \cos\left(2\pi f_{0}t\right),$$

and the output waveform of the signal after the unmatched filter is

(3) $$y_{2}\left(t\right)=\frac{1}{\sqrt{BT_{b}}}\exp\left(\frac{j\pi \mu t^{2}}{2}\right)\left[C\left(X\right)+jS\left(X\right)\right],$$

where $X=\frac{\pi }{2}\left(\sqrt{BT_{b}}-\sqrt{\mu }{\left|t\right|}^{2}\right)$, C(·), and S(·) are the standard Fresnel cosine and sine integrals, respectively, and are given by

(4) $$C\left(x\right)=\int_{0}^{x}cos\left(\frac{\pi }{2}t^{2}\right)dt,\;S\left(x\right)=\int_{0}^{x}sin\left(\frac{\pi }{2}t^{2}\right)dt.$$

As shown in Equation (2), the output of the matched filter has similar characteristics to the sinc function, which can convert the input chirp signal with low peak power into an output signal with high peak power and concentrated energy in a very short period of time. The feature is beneficial for detecting chirp signals. Finally, the information bit can be restored by sampling and judging the output of the matched filter.

### 2.2. Troposcatter Channel Model

The main characteristics of tropospheric scattering propagation encompass transmission loss and fading. Transmission loss is influenced by various factors, such as the distance between the transmitter and receiver, the geographical location, and the scattering properties of scatterers. There are some classical methods for estimating scattering transmission losses, mainly the NBS-101 model and ITU-R model.

NBS-101 is a widely used calculation method to estimate transmission losses in tropospheric scatter communication links. The NBS-101 prediction method considers the impact of nonlinear variations in the atmospheric refraction index when utilizing the equivalent earth radius. It appropriately adjusts this factor during the calculation process to align with actual link conditions, resulting in a prediction of scattering transmission loss that is more consistent with measured values. However, this method involves complex calculations and requires consultation of numerous charts, making its engineering application relatively challenging.

The ITU-R P.617 model proposes the data and propagation prediction techniques required for the design of a radio relay system beyond the horizon, as well as a transmission loss prediction model for troposcatter signals. Many changes have been made since the P.617 recommendation was first proposed in 1992. From P.617-1 in 1992 to P.617-3 in 2013, this model was mainly used to predict the performance of a radio relay system from 50% to 99.999% of the time percentage. As it was updated to P.617-4 in 2017 and P.617-5 in 2019, the model was improved to predict performance for a time percentage of 0.001% to 99.999%. This pattern can be expressed as follows [24]:

(5) $$L=F+22\log f+35\log\theta +17\log d+L_{c}-Y_{p},$$

In Equation (5), *L* denotes the median of transmission loss (dB), *F* is weather factor (dB), *f* stands for frequency (MHz), θ is the scatter angle (mrad), *d* is the circuitry length (km), $L_{c}$ is the surface medium coupling loss (dB), and $Y_{p}$ is the conversion factor that does not exceed the estimated *p* time percentage.

The calculation process of the ITU-R617 prediction method is relatively simple, and most of the unknowns can be obtained through the corresponding calculation formula, accurately calculating the transmission loss of tropospheric scattering communication, which is more in line with the practical applications in China.

Using the tranmission loss, as computed in Equation (5), the receiver signal-to-noise ratio (SNR) γ can be expressed as [25]

(6) $$\gamma =10\log_{10}P_{t}-L-L_{F}-NF-10\log_{10}\left(KT_{1}B\right),$$

where $P_{t}$ is the transmitter power, $L_{F}$ is the transmitter and receiver feeder losses, NF is the receiver noise figure, *K* is Boltzman’s constant, $T_{1}$ is the noise temperature in Kelvin, and *B* is the transmission bandwidth.

Fading is a typical characteristic of tropospheric scattering signals, which can be classified into fast fading and slow fading according to the duration of the fading period [26]. Slow fading reflects the attenuation caused by changes in the average value of a received level over a medium range of hundreds of wavelengths, primarily associated with climate conditions. Statistical findings indicate that the slow fading signal power obeys a log-normal distribution. Fast fading comprises frequency-selective fading and time-selective fading, which reflects the fast amplitude changes over a short period of time. The statistical experimental results show that fast fading always follows the Rayleigh distribution [27]. The received SNR γ will be used to analyze and simulate modulation performance over a troposcatter fading channel.

The troposcatter channel is commonly referred to as Wide-Sense Stationary Uncorrelated Scattering (WSSUS), which follows Rayleigh distributions [28]. It is a linear time-varying (LTV) system that can be characterized as a continuous time–impulse response $\tilde{c}\left(\tau ,t\right)$ [29]. Assuming that the bandwidth of the low-pass input of the channel is limited to the order of r/2, and under the band-pass condition, it is equal to B=r, where *r* represents the symbol rate. Therefore, the low-pass input signal is sampled at a minimum sampling rate of *r* points per second, with a sampling interval represented by T=1/r, i.e., tap delay.

With transmit signal $\tilde{x}\left(t\right)$ input, the output $\tilde{y}\left(t\right)$ of the troposcatter multipath channel with the tap delay line model (TDL) can be described as

(7) $$\tilde{y}\left(t\right)=T\sum_{n=0}^{N-1}\tilde{x}\left(t-nT\right)\tilde{c}\left(nT,t\right)=\sum_{n=0}^{N-1}\tilde{x}\left(t-nT\right){\tilde{g}}_{n}\left(t\right),$$

where ${\tilde{g}}_{n}\left(t\right)=T\tilde{c}\left(nT,t\right)$, *N* denotes the number of taps in the TDL model, i.e., the number of multipaths in the scattering channel.

For the Rayleigh fading channel, ${\tilde{g}}_{n}\left(t\right)$ is the tap gain, which is a complex Gaussian process with a zero mean [30]. Under the WSSUS assumption, the gain of each tap is uncorrelated, their power spectral density is determined by the Doppler spectrum, and the magnitude of the Doppler shift reflects the rate at which the tap coefficient changes.

In consideration of the aforementioned characteristics of the scattering channel, and its multipath structure that is always consistent with the Rayleigh distribution, an equal-delay multipath Rayleigh fading model is employed for the simulation of non-anomalous propagation within the scattering channel. In this paper, the seven-path Rayleigh fading channel model with equal delay is adopted for the purposes of simulation analysis.

### 2.3. Chirp-BOK System Model

**Figure 2:**
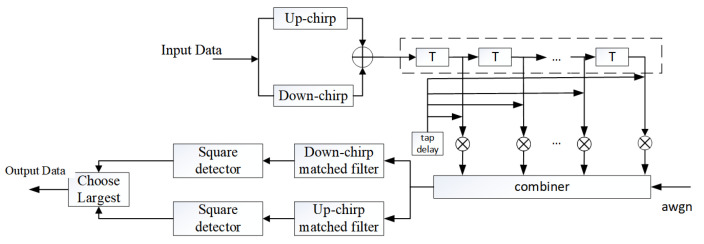
Scattering communication system based on chirp-BOK modulation.

Chirp-BOK uses a quasi-orthogonal chirp signal with up- and down-frequency to carry different information bits. The up-chirp signal represents the bit “1”, and the down-chirp signal represents the bit “0”. After passing through the communication channel, the receiver uses a down-chirp and up-chirp matched filter to detect and judge for demodulation. The schematic diagram of the chirp-BOK modulation system is shown in Figure 2 and Algorithms 1 and 2.
**Algorithm 1:** Chirp-BOK modulation algorithm.
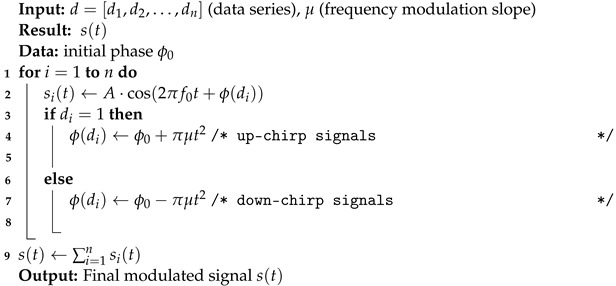


**Algorithm 2:** Chirp-BOK demodulation algorithm.

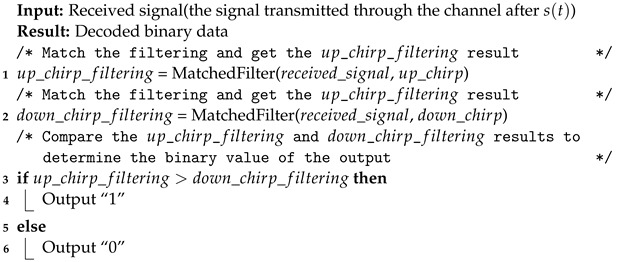



## 3. Theoretical Analysis for Bit Error Rate

### 3.1. Rayleigh Flat Fading

The average bit error rate (BER) (${\bar{p}}_{e}$) of chirp-BOK modulation over a flat fading channel can be calculated by the instantaneous BER integrated over an AWGN channel $p_{e}\left(\gamma \right)$ with a signal-to-noise ratio (SNR) γ as follows:

(8) $${\bar{p}}_{e}=\int_{0}^{\infty }p_{e}\left(\gamma \right)f_{\gamma }\left(\gamma \right)d\gamma ,$$

where $p_{e}\left(\gamma \right)$ is given by [31]

(9) $$P_{e}\left(\gamma \right)=Q\left(\sqrt{\frac{E_{b}}{N_{0}}\left(1-\rho_{ij}\right)}\right)=\frac{1}{2}erfc\left(\sqrt{\frac{1}{2}\gamma }\right).$$

where $E_{b}$ is symbol energy and $N_{0}$ is noise power spectral density. The Gaussian Q-function is defined by $Q\left(x\right)=\frac{1}{\sqrt{2\pi }}\int_{x}^{\infty }e^{-t^{2}/2}dt$, and $\rho_{ij}$ is the normalized cross-correlation coefficient between the up/down-chirp signals, as follows:

(10) $$\rho_{ij}=\frac{1}{\sqrt{BT_{b}}}\sqrt{{\left(C\left(\frac{\pi }{2}BT_{b}\right)\right)}^{2}+{\left(S\left(\frac{\pi }{2}BT_{b}\right)\right)}^{2}}.$$

For Rayleigh fading, the probability density function (PDF) of the received SNR γ and the cumulative distribution function (CDF) of the received SNR γ can be described, respectively, as

(11) $$\begin{array}{cc} f_{\gamma }\left(\gamma \right) & =\frac{1}{\bar{\gamma }}exp\left(\frac{-\gamma }{\bar{\gamma }}\right), \end{array}$$

(12) $$\begin{array}{cc} F_{\gamma }\left(\gamma \right) & =1-exp(\frac{-\gamma }{\bar{\gamma }}), \end{array}$$

where $\gamma =\frac{{\left|h\right|}^{2}E_{b}\left(1-\rho_{ij}\right)}{N_{0}}$ is the instantaneous SNR per bit, *h* is the fading coefficient with the Rayleigh distribution, and $\bar{\gamma }=\frac{{\left|\bar{h}\right|}^{2}E_{b}\left(1-\rho_{ij}\right)}{N_{0}}$ is the average SNR per bit. Additionally, since Equation (9) is a complementary error function, its original function cannot be easily expressed by a simple elementary function, so the integral by parts method is used to calculate Equation (8). Thus, Equation (8) can be deduced by

(13) $${\bar{p}}_{e}=-\int_{0}^{\infty }p_{e}^{\prime }\left(\gamma \right)F_{\gamma }d\gamma ,$$

where

(14) $$p_{e}^{\prime }\left(\gamma \right)=-\sqrt{\frac{1}{8\pi }}{\left(\gamma \right)}^{-\frac{1}{2}}exp\left(-\frac{1}{2}\gamma \right).$$

By substituting Equations (12) and (14) into Equation (13), we can get

(15) $${\bar{p}}_{e}=\frac{1}{2}\left(1-\sqrt{\frac{\bar{\gamma }}{2+\bar{\gamma }}}\right)$$

Hence, the average BER of the chirp-BOK modulation over the Rayleigh flat fading channel can be written in a closed form as

(16) $${\bar{p}}_{e}=\frac{1}{2}\left(1-\sqrt{\frac{{\bar{h}}^{2}E_{b}\left(1-\rho_{ij}\right)/N_{0}}{2+{\bar{h}}^{2}E_{b}\left(1-\rho_{ij}\right)/N_{0}}}\right).$$

### 3.2. Frequency-Selective Fading

The impulse response of baseband signals on frequency-selective channels can be expressed as

(17) $$h\left(t\right)=\sum_{l=1}^{L}\alpha_{l}e^{-j\theta_{l}}\delta \left(t-\tau_{l}\right),$$

where *l* is the channel index, *L* is the number of resolvable paths, $\delta \left(\cdot \right)$ is the delta function, and ${\left\{\alpha_{l}\right\}}_{l=1}^{L}$, ${\left\{\theta_{l}\right\}}_{l=1}^{L}$, ${\left\{\tau_{l}\right\}}_{l=1}^{L}$ are the random channel amplitudes, phases and delays, respectively. The received signal can be expressed as

(18) $$\begin{array}{cc} y\left(t\right) & =h\left(t\right)*s\left(t\right)+n\left(t\right)+i_{ISI}\left(t\right)\\ \; & =\sum_{l=1}^{L}\alpha_{l}e^{-j\theta_{l}}x\left(t-\tau_{l}\right)+n\left(t\right)+i_{ISI}\left(t\right), \end{array}$$

where $n\left(t\right)$ denotes Additive White Gaussian Noise (AWGN) and $i_{ISI}\left(t\right)$ is the inter symbol interference (ISI) component. Assuming that the fading amplitude is a random variable with a mean-square value $\Omega =\bar{\alpha^{2}}$. The instantaneous SNR per bit of the Lth channel is given as $\gamma_{l}=\Omega_{l}E_{b}\left(1-\rho_{ij}\right)/N_{0}$ and the average SNR per bit of the Lth channel can be expressed as ${\bar{\gamma }}_{l}={\bar{\Omega }}_{l}E_{b}\left(1-\rho_{ij}\right)/N_{0}$.

The mean-square value $\Omega_{l}$ is related to power delay profile of the channel, which is considered as an exponentially decaying power delay profile (PDP).

(19) $$\Omega_{l}=\Omega_{1}e^{-\left(l-1\right)\delta },\delta \geq 0,l=1,2,\ldots ,L,$$

where $\Omega_{1}$ is the average fading power of the first path, and the parameter δ is the power decay factor. The variance of the ISI component is [32]$\sigma_{ISI}^{2}=\left(\Omega_{T}-1\right)\Omega_{1}E_{b}/2P_{g}$, where $\Omega_{T}=\sum_{l=1}^{L}e^{-\left(l-1\right)\delta }$ is the normalized total average fading power, and $p_{g}$ is the processing gain. Therefore, the equivalent interference plus noise power spectral density can be written as

(20) $$\frac{N_{e}}{2}=\frac{\Omega_{T}-1}{2P_{g}}\Omega_{1}E_{b}+\frac{N_{0}}{2}$$

The average signal-to-interference plus noise ratio is

(21) $${\bar{\gamma }}_{e}={\left(\frac{2}{{\bar{\gamma }}_{1}}+\frac{\Omega_{T}-1}{2P_{g}}\right)}^{-1}.$$

Following the same procedure as in Section 3, the average BER over frequency-selective Rayleigh fading in a closed form is obtained as

(22) $${\bar{p}}_{e}=\frac{1}{2}\left[1-\sqrt{\frac{{\bar{\gamma }}_{e}}{1+{\bar{\gamma }}_{e}}}\right].$$

## 4. Simulation and Discussion

In the subsequent simulation, the setting parameters of the multipath Rayleigh fading channel are shown in Table 1.

**Figure 3:**
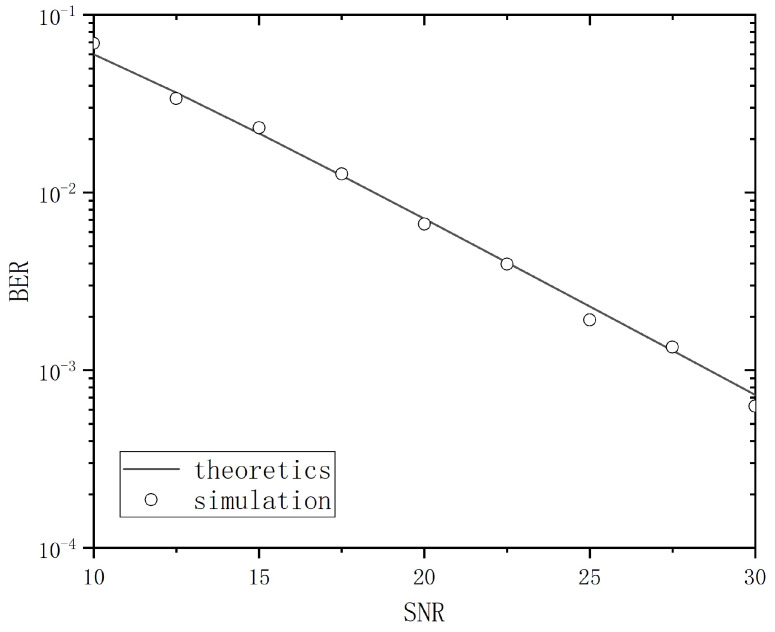
Comparison of theoretical and simulated values.

Equation (14) states the average BER output from the troposcatter channel with chirp-BOK modulation in the absence of ISI (i.e., Rayleigh flat fading), which can be expressed as $P_{e}=\frac{1}{2}\left(1-\sqrt{\frac{\bar{\gamma }}{2+\bar{\gamma }}}\right)$, where $\bar{\gamma }$ denotes the average SNR. A comparison between the simulation result and theoretical result is shown in Figure 3; the simulation results were verified through numerous simulations, revealing that when the parameter *D* is set to 6, there is a high degree of consistency with the theoretical results.

**Figure 4:**
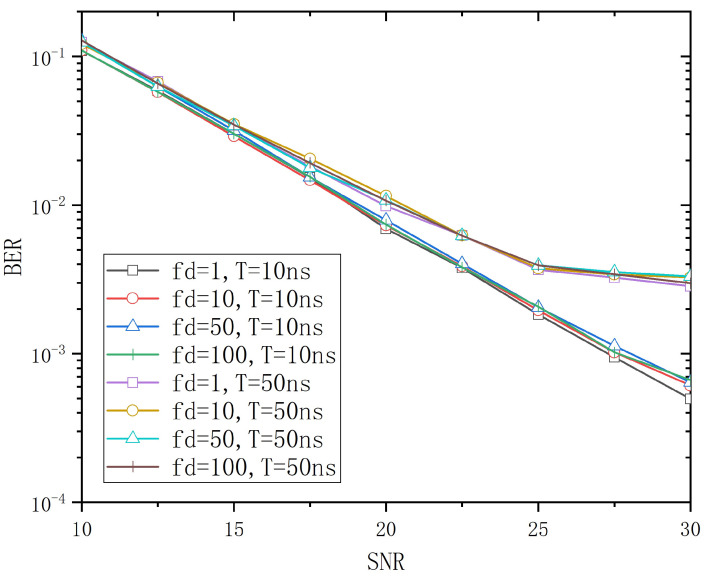
The BER curve of different $f_{d}$ in T=10ns and T=50ns.

When tap delay *T* is equal to 10ns and 50ns, respectively, with Path-Gain-Vector1, the influence of different Doppler shifts $f_{d}$ on the system BER performance is shown in Figure 4. As shown in Figure 4, the BER curves are basically consistent in the range of $f_{d}\in \left[1\;\mathrm{Hz},100\;\mathrm{Hz}\right]$, and the influence of $f_{d}$ on the BER is relatively small. The subsequent simulation and analysis are based on the assumption that $f_{d}=10\;\mathrm{Hz}$.

**Figure 5:**
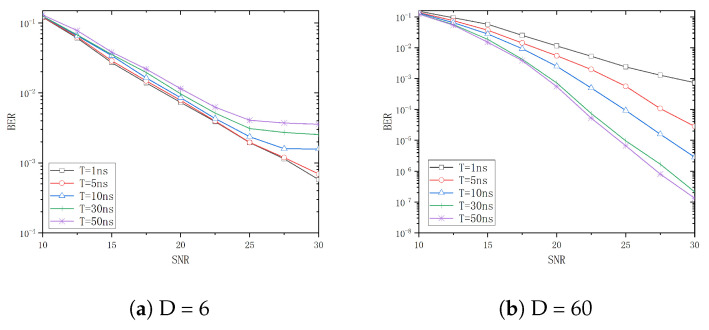
The BER curve of different *T*.

The effect of varying tap delay vector *T* on BER performance of the system with *D* equal to 6 and 60 is illustrated in Figure 5a and Figure 5b, respectively.

As illustrated in Figure 5a, under the premise of a small *D*, the BER curve exhibits a flattening trend as the value of *T* increases. When T=1ns and T=ns, the two BER curves are essentially identical, indicating that the ISI in the system is small in the presence of a relatively short time interval. This can be regarded as frequency flat fading. As the value of *T* increases to 10ns, 30ns, and 50ns, the BER performance of the system declines continuously. This is due to the fact that as *T* increases, the ISI phenomenon in the channel becomes more pronounced, resulting in a transition from a frequency flat fading channel to a frequency-selective fading channel.

Figure 5b shows that the BER performance improves with an increase in *T*. This is due to the fact that a larger time–bandwidth product results in a more rectangular spectral characteristic of the chirp signal, rendering it more resistant to frequency-selective fading. Consequently, the system is more effectively capable of suppressing frequency-selective fading caused by different paths when *T* continuously increases, thus enhancing the reception quality and BER performance.

**Figure 6:**
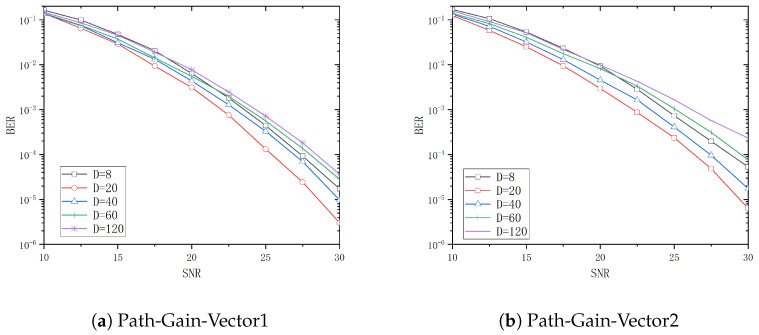
The BER curve of different *D* in T=5ns.

**Figure 7:**
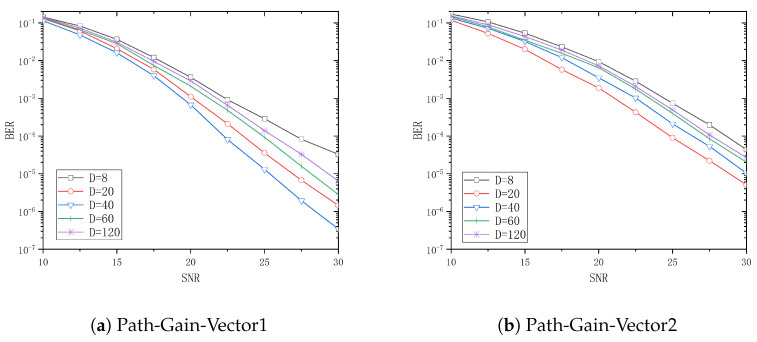
The BER curve of different *D* in T=10ns.

**Figure 8:**
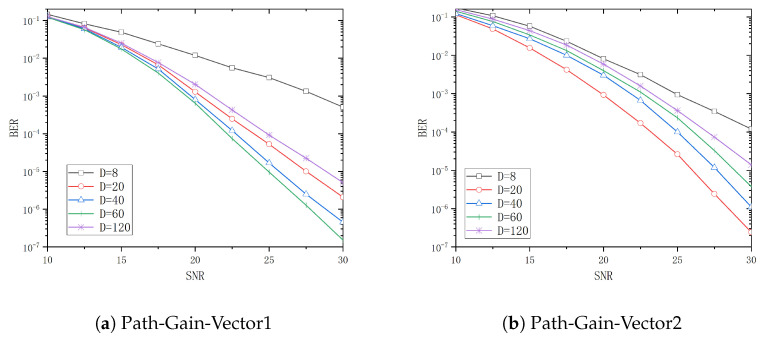
The BER curve of different *D* in T=30ns.

**Figure 9:**
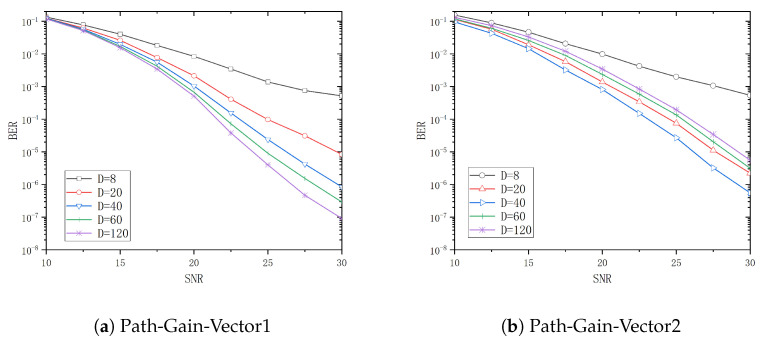
The BER curve of different *D* in T=50ns.

The system BER performance for tap delays *T* of 5ns, 10ns, 30ns, and 50ns is analyzed in Figure 6, Figure 7, Figure 8 and Figure 9 when *D* is 8, 20, 40, 60, and 120, respectively. From these figures, it can be observed that the system performance attains its optimal level when *D* is 20, 40, 60, and 120, respectively, with Path-Gain-Vector1. Similarly, the system performance is optimal when *D* is 20 and 40 with Path-Gain-Vector2.

Table 2 and Table 3 show the threshold values of the SNR for different channel delay vectors and different *D* when the BER is $10^{-4}$ with Path-Gain-Vector1 and Path-Gain-Vector2, respectively.

Assume that target bit error rate is $10^{-4}$ and the reference D=8 is used as a basis for the gain calculation. In Path-Gain-Vector1, the optimal selection to achieve the target BER is 1.7dB less than the SNR required for D=8 when the tap delay *T* is 5ns. Similarly, the optimal selections to achieve the target BER are 5dB, 9.5dB and 10.0dB less than D=8 when the tap delay *T* is 10ns, 30ns and 50ns, respectively. In Path-Gain-Vector2, the optimal configuration to reach the target BER is 1.7dB less than the SNR required for D=8 when the tap delay *T* is 5ns. Similarly, the optimal configurations to reach the target BER are 3.9dB, 7.7dB, and 9.8dB less than D=8 when the tap delay *T* is 10ns, 30ns and 50ns, respectively.

**Figure 10:**
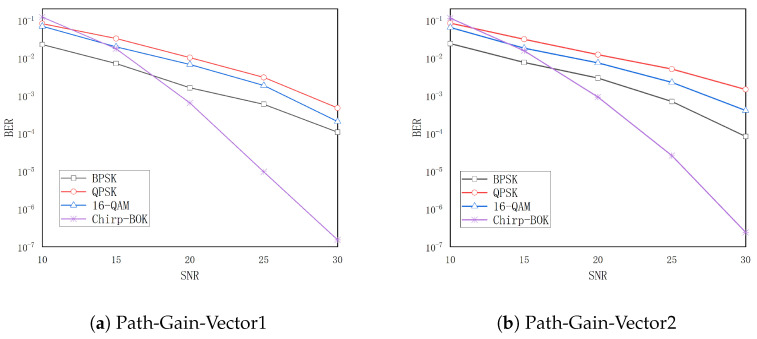
The BER curve of different modulation schemes in T=30ns.

Figure 10 is a BER performance comparison between chirp-BOK, BPSK, QPSK and 16-QAM modulation schemes. During the chirp-BOK process of simulation, the BER trends were found to be consistent under four different channel conditions. Therefore, only two cases are listed here, T=30ns and various path gains, with results showing that chirp-BOK outperforms BPSK, 16-QAM and QPSK in troposcatter deep fading channel conditions. Although chirp-BOK modulation can have higher sensitivity, the spectral efficiency is relatively low. To settle this problem, the chirp signal spread method can be used with a typical digital modulation scheme and Orthogonal Frequency Division Multiplexing (OFDM) technique.

## 5. Conclusions

In this paper, we presented a BER performance analysis and parameter optimization of chirp-BOK modulation over a troposcatter channel, theoretically and numerically. Firstly, the averaged BER performance of chirp-BOK modulation over a Rayleigh flat fading channel was derived theoretically. Then, a seven-path equal-delay Rayleigh fading channel model was established to simulate the chirp-BOK modulation BER performance under various conditions. Furthermore, the impact of the time–bandwidth product and channel tap delay was optimized and analyzed with different chirp-BOK parameters. These results demonstrate that when the path gain remains constant and the tap delay increases, the value of *D* needs to be increased in order to achieve optimal performance. It is worth noting that an increase in the primary path gain will not necessarily result in an improvement in BER performance, when the same time–bandwidth product and tap delay conditions are considered. As observed in Part 4, when optimized with appropriate parameters, chirp-BOK modulation can exhibit a significantly lower BER compared to other modulation techniques under deep fading channel conditions. On the one hand, in a future study, an actual chirp signal experiment should be tested to characterize chirp-BOK modulation parameters. On the other hand, further enhancement may focus on the combination of chirp modulation with some advanced error correction codes to achieve higher data transmission reliability.

## Figures and Tables

**Table 1:** Parameters for simulation.

Parameter	Value
Maximum Doppler Shift	$f_{d}\in \left[1\;\mathrm{Hz},100\;\mathrm{Hz}\right]$
Delay-Vector	$\left[0\;1\;2\;3\;4\;5\;6\right]\times T\left(\mathrm{ns}\right)$
Path-Gain-Vector1	$\left[-10\;-30\;-2\;-5\;-8\;-10\right]\;\left(\mathrm{dB}\right)$
Path-Gain-Vector2	$\left[10\;-30\;-2\;-5\;-8\;-10\right]\;\left(\mathrm{dB}\right)$
Time–Bandwidth Product	$D=BT_{b}$

**Table 2:** SNR $\left(dB\right)$ for Path-Gain-Vector1 when the BER is $10^{-4}$.

Path-Gain-Vector1	T=5ns	T=10ns	T=30ns	T=50ns
D=8	27.2	27.5	31.9	32.5
D=20	25.5	24.0	23.5	25.0
D=40	26.8	22.5	22.8	22.7
D=60	28.2	25.0	22.4	22.5
D=120	28.8	25.8	25.0	21.4

**Table 3:** SNR $\left(dB\right)$ for Path-Gain-Vector2 when the BER is $10^{-4}$.

Path-Gain-Vector2	T=5ns	T=10ns	T=30ns	T=50ns
D=8	28.7	28.7	30.4	32.7
D=20	26.0	24.8	22.7	23.4
D=40	27.5	26.9	25.1	22.9
D=60	29.1	27.4	26.3	25.4
D=120	32.6	27.7	27.4	25.6

## Data Availability

Data are contained within this article.

## Abbreviations

The following abbreviations are used in this manuscript:

CSS	Chirp spread spectrum
BOK	Binary orthogonal keying
ISI	Inter-symbol interference
BER	Bit error rate
SNR	Signal-to-noise power ratio
PDF	Probability density function
CDF	Cumulative distribution function
